# Supplementary material for: Single-molecule dynamics reveal ATP binding alone powers substrate translocation by an ABC transporter
Source: Nat Commun. 2026 Mar 31;17:3038. doi: 10.1038/s41467-026-70021-1 (PMC13040083; doi:10.1038/s41467-026-70021-1)
Supplement: Supplementary file 1 — Supplementary Information [file 41467_2026_70021_MOESM1_ESM.pdf]

Single-molecule dynamics reveal ATP binding alone powers substrate translocation by an ABC transporter

Christoph Nocker[1], Matija Pečak[1], Tobias Nocker[1], Amin Fahim[1], Lukas Sušac[1], Robert Tampé[1,*]

[1] Institute of Biochemistry, Biocenter, Goethe University Frankfurt, Max-von-Laue Str. 9, Frankfurt a.M., Germany

*Corresponding author: tampe@em.uni-franfurt.de

Content:

Supplementary Fig. 1-17

Supplementary Table 1

Supplementary Figures

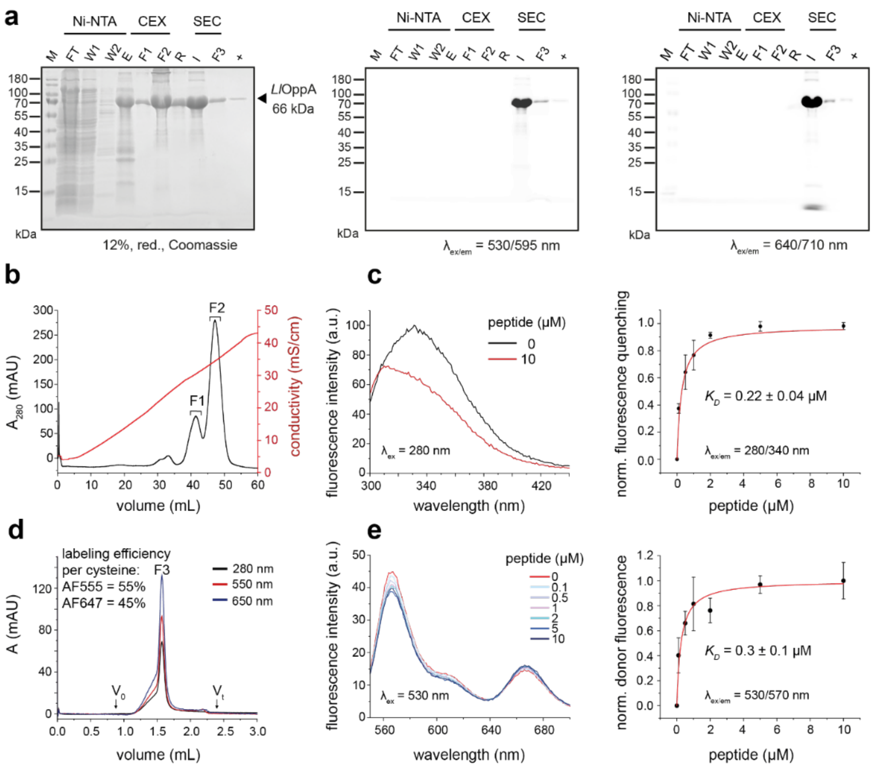

Supplementary Fig. 1 | Purification, labeling, and peptide-binding properties of the FRET sensor. a, Left: SDS-PAGE (12% (w/v) acrylamide, reducing; Coomassie-stained) showing the purity of *Ll*OppA after Ni-NTA metal affinity and cation exchange chromatography (CEX). Middle and right: in-gel fluorescence of donor and acceptor-labeled *Ll*OppA before and after size-exclusion chromatography (SEC). Lane labels: M, molecular weight marker; FT, flow-through; W1, W2, wash steps 1 and 2; F1-F3, collected fractions; R, refolded *Ll*OppA; I, SEC input; pos. cont., positive control. **b**, CEX chromatography corresponding to factions F1 and F2 shown in (**a**). **c**, Left: Tryptophan fluorescence emission spectra of unlabeled *Ll*OppA in the absence and presence of a saturating peptide (RRYQKSTEL). Right: peptide-binding affinity of unlabeled *Ll*OppA for RRYQKSTEL measured via tryptophan fluorescence quenching, yielding a dissociation constant (K_D) of 0.22 ± 0.04 µM (mean ± s.d., n = 3 independent experiments). **d**, SEC profile of AF555/AF647-labeled *Ll*OppA^{AS} (hereafter OppA) used to determine labeling efficiencies: 55% for AF555 and 45% for AF647 per cysteine. Fraction F3 corresponds to the SDS-PAGE fraction in (**a**). **e**, Left: Ensemble FRET spectra of OppA in response to increasing peptide concentrations. Right: Quantification of three ensemble FRET experiments to determine peptide-binding affinity, yielding a K_D of 0.3 ± 0.1 µM (mean ± s.d., n = 3 independent experiments).

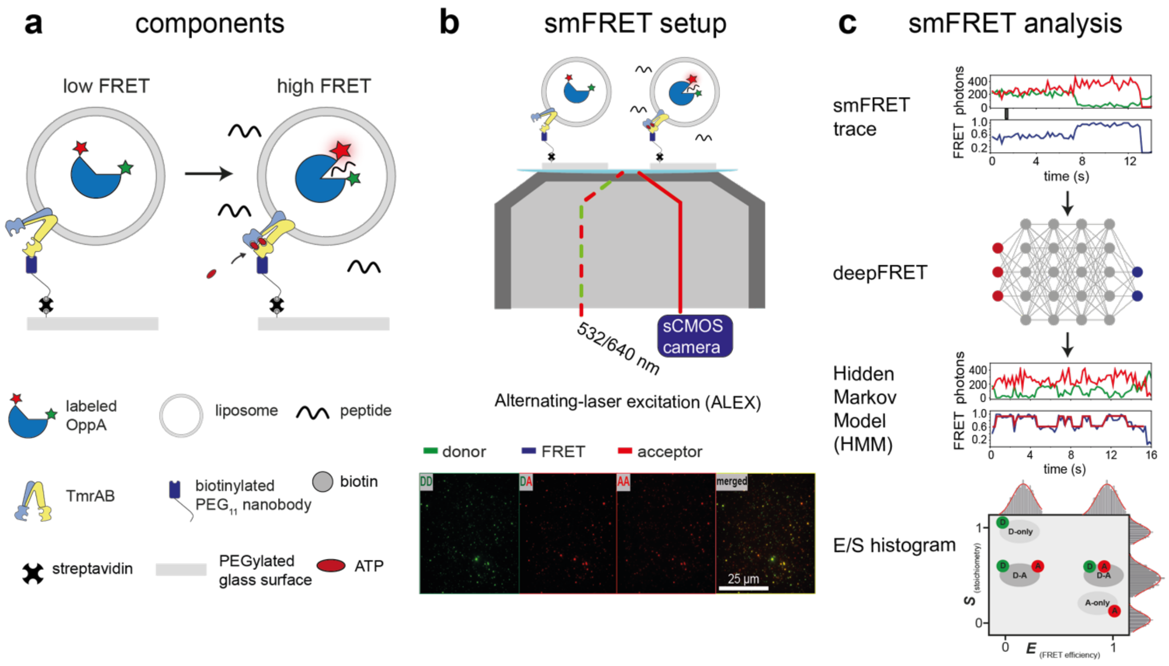

Supplementary Fig. 2 | Schematic of the smFRET setup. a, Scheme of the single-molecule Förster resonance energy transfer (smFRET) setup showing the immobilization strategy. Individual TmrAB transport complexes reconstituted in liposomes were captured on a passivated surface via a biotinylated PEG_{11}-conjugated nanobody. **b**, Total internal reflection fluorescence (TIRF) microscopy with alternating laser excitation (ALEX) was used for single-molecule imaging. This setup enables selective excitation of donor and acceptor fluorophores and determination of fluorophore stoichiometry. Representative fluorescence micrographs show donor emission upon donor excitation (DD), acceptor emission upon donor excitation (DA), and a merged image highlighting colocalized donor and acceptor signals. **c**, Overview of the smFRET data analysis workflow. Fluorescence traces were analyzed using DeepFRET, and only traces with ≥80% confidence were included for histogram generation and dwell time analysis of dynamic transitions.

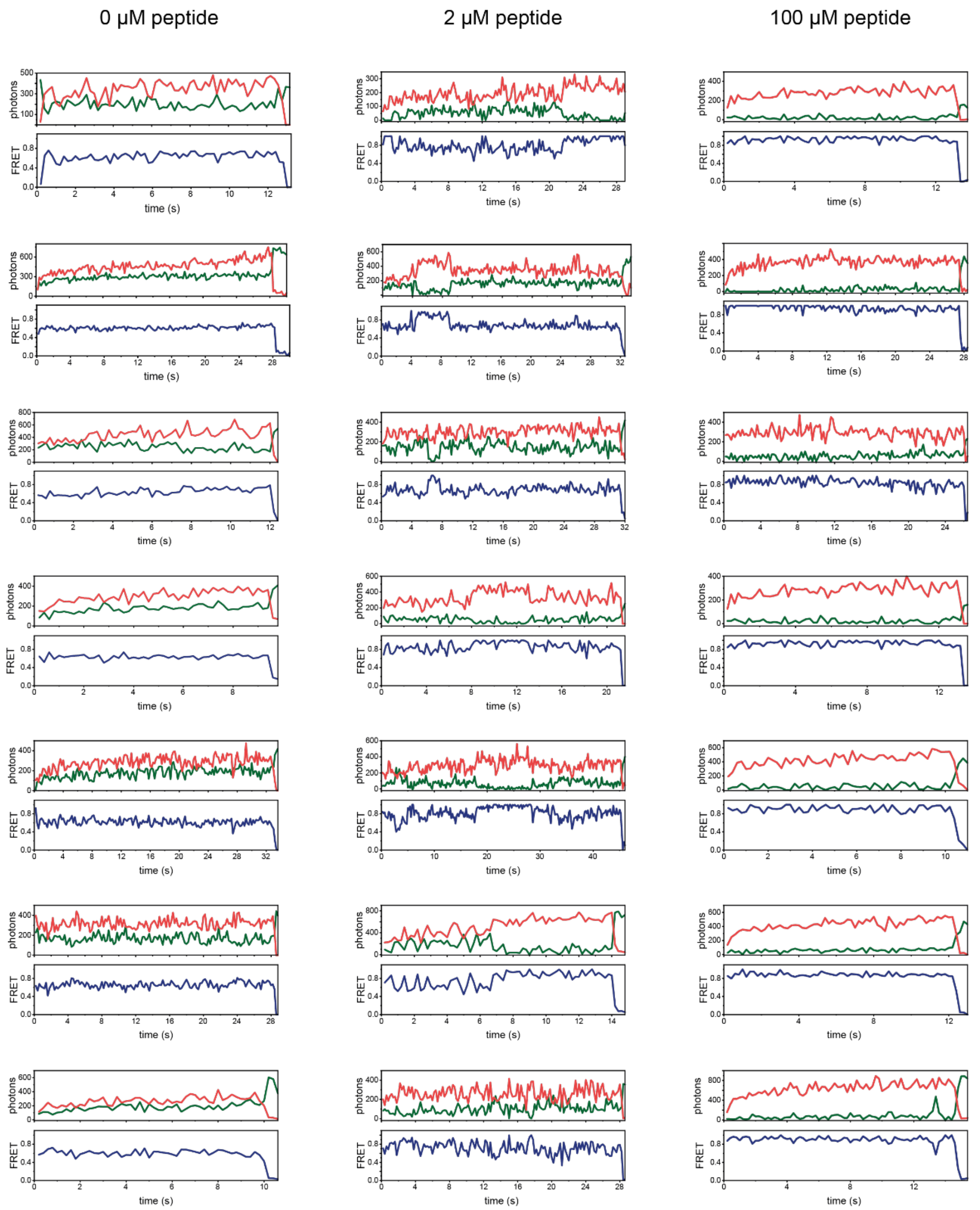

Supplementary Fig. 3 | smFRET traces of OppA. Representative smFRET traces of OppA under different peptide conditions. Left: OppA in the absence of the peptide showing predominantly low-FRET states. Middle, OppA at 2 µM peptide concentration exhibiting dynamic transitions between low- and high-FRET states. Right, OppA at 100 µM peptide concentration showing stable high-FRET states, indicative of saturating peptide binding.

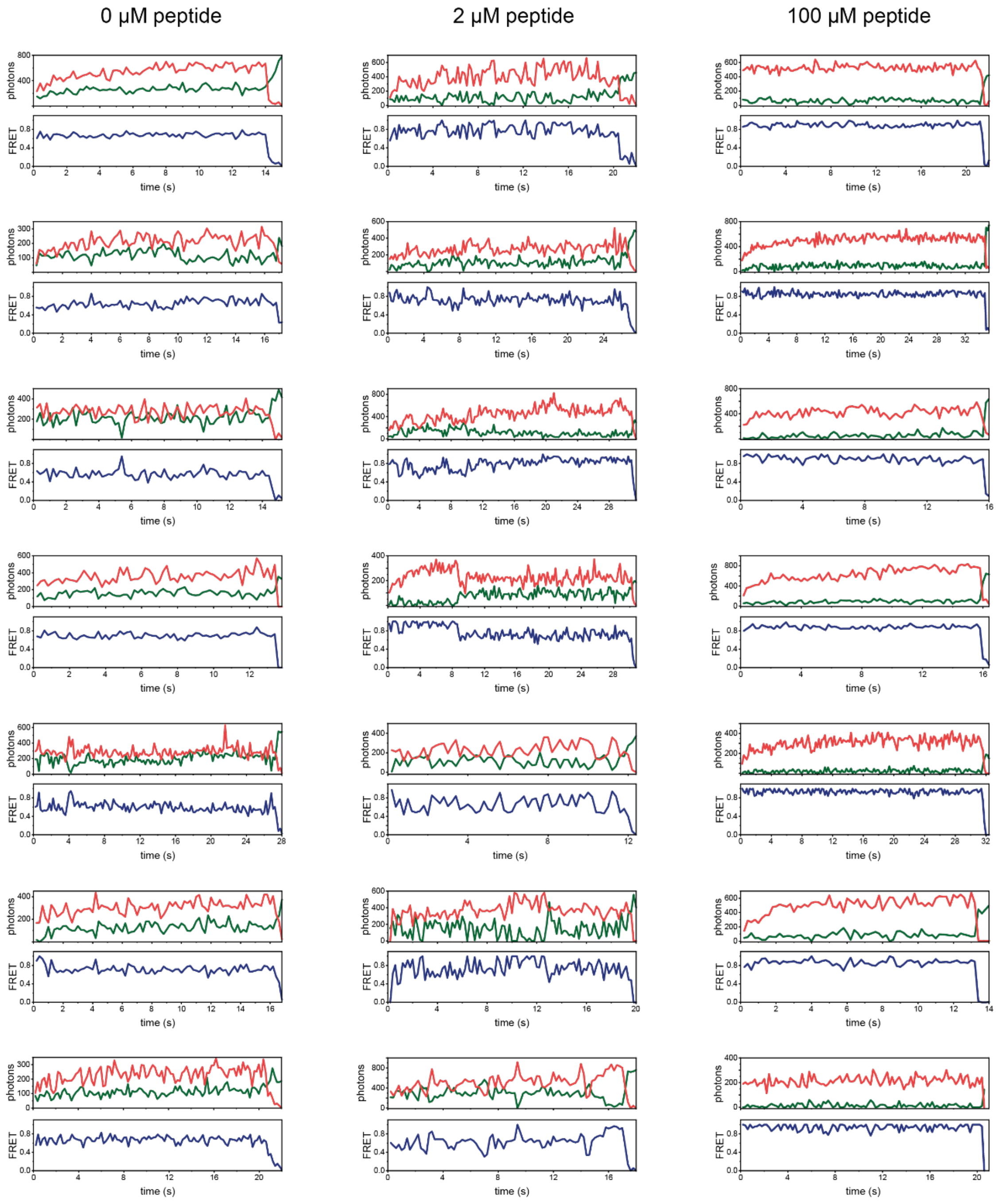

Supplementary Fig. 4 | smFRET traces of OppA (continuation). Representative smFRET traces of OppA under different peptide conditions. Left: OppA in the absence of the peptide showing predominantly low-FRET states. Middle, OppA at 2 µM peptide concentration exhibiting dynamic transitions between low- and high-FRET states. Right, OppA at 100 µM peptide concentration showing stable high-FRET states, indicative of saturating peptide binding.

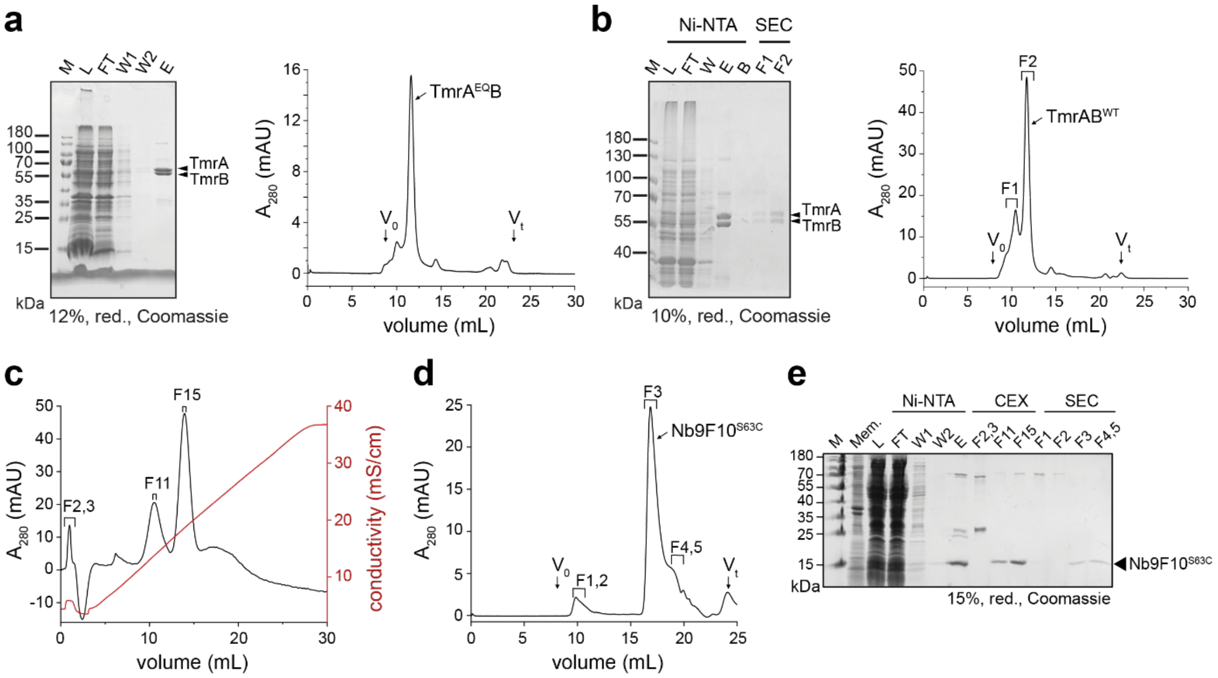

Supplementary Fig. 5 | Purification of TmrA^{EQ}B, TmrAB^{WT}, nanobody Nb9F10^{S63C}, and formation of TmrAB-nanobody complexes. **a**, Left: SDS-PAGE (12% (w/v) acrylamide, reducing; Coomassie-stained) showing purified TmrA^{EQ}B after metal affinity chromatography (Ni-NTA). Lanes: M, molecular weight marker; L, load; FT, flow-through; W1 and W2, wash steps 1 and 2; E, eluate. Right: Size-exclusion chromatography (SEC) profile of TmrA^{EQ}B, with the monodisperse peak fraction collected for subsequent experiments. **b**, Left: SDS-PAGE (10% (w/v) acrylamide, reducing; Coomassie-stained) of purified TmrAB^{WT} after Ni-NTA chromatography. Lanes: B, Ni-NTA agarose beads; F1 and F2, SEC fractions. Right: SEC profile of TmrAB^{WT}. Fraction F2 was selected for subsequent experiments, although both fractions containing TmrAB in the same purity. The double-peak is based on the transporter per DDM micelle ratio (F1: two TmrAB per micelle; F2: one TmrAB per micelle). **c**, Cation exchange chromatography (CEX) profile of the single-cysteine nanobody variant Nb9F10^{S63C}. **d**, SEC profile of pooled CEX fractions from (c). **e**, SDS-PAGE (15% (w/v) acrylamide, reducing; Coomassie-stained) confirming the purity of Nb9F10^{S63C} following Ni-NTA, CEX, and SEC purification steps.

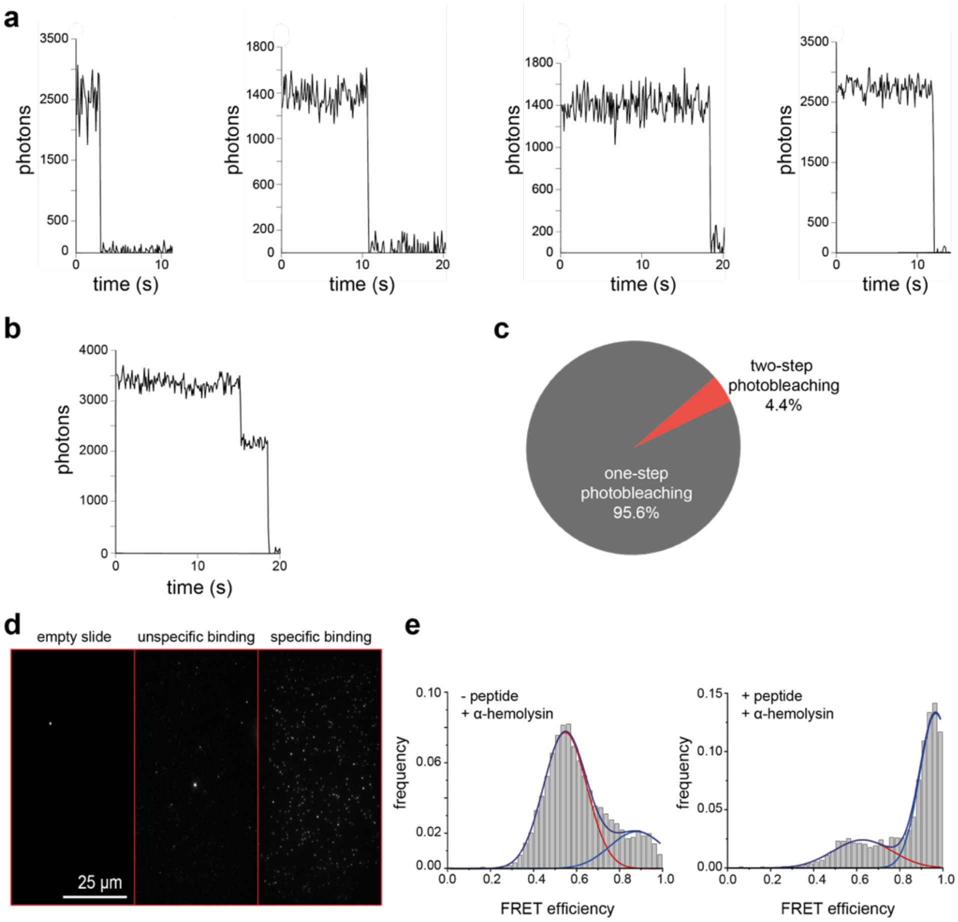

Supplementary Fig. 6 | Quantification of stochastic TmrAB reconstitution and assay validation. a, **b**, AF647-labeled TmrAB^{WT}, containing a single cysteine for site-specific Alexa Fluor 647 (AF647) labeling, was reconstituted into liposomes at a protein-to-lipid ratio of 1:10,000 (w/w). Representative fluorescence trajectories show either (**a**) single-step or (**b**) two-step photobleaching, corresponding to one or two transporters per liposome, respectively. **c**, Analysis of n = 2088 photobleaching traces demonstrates that only 4.4% of fluorescent liposomes exhibit two photobleaching steps, whereas the vast majority contain a single transporter (95.6%). **d**, Control experiments assessing liposome binding specificity. Left: PEGylated glass surface incubated with streptavidin in the absence of liposomes. Middle: Liposomes containing TmrAB and OppA applied without biotinylated PEG_{11}-nanobody, showing minimal nonspecific binding. Right: Specific immobilization of TmrAB/OppA-containing liposomes via biotinylated PEG_{11}-nanobody. **e**, Peptide uptake assay validating sensor functionality. Peptide RRYQKSTEL (200 µM) was translocated into OppA-containing liposomes via α-hemolysin (2 µM). Left: FRET efficiency histogram of apo state (low FRET, red; E = 0.6, n = 65). Right: Histogram of the holo state (high FRET, blue; E = 0.9, n = 88).

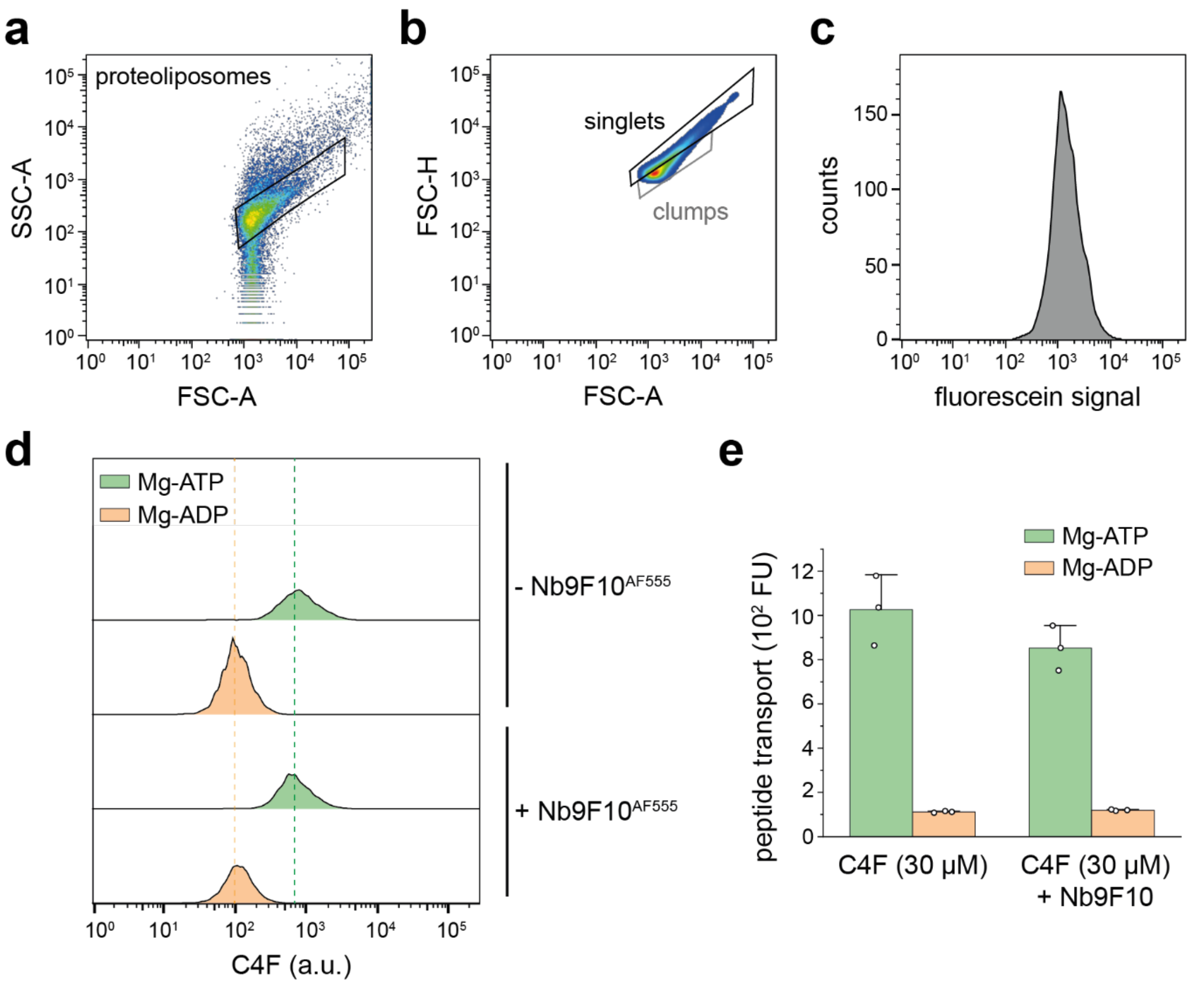

Supplementary Fig. 7 | Nanobody binding does not impair TmrAB transport activity. **a**, Single-liposome transport assay analyzed by flow cytometry. Gating strategy used to identify TmrAB-containing liposomes labeled with Nb9F10$^{S63C\text{-}AF555}$. Liposomes lacking nanobody fluorescence were excluded from analysis in the TmrAB-nanobody sample. Proteoliposomes were gated on characteristic forward scatter (FSC) and side scatter (SSC) signals. **b**, Doublet discrimination to ensure analysis of single liposomes. **c**, Fluorescence signal from transported peptide RRYC^{Fluorescein}KSTEL (C4F) in TmrAB-containing liposomes, recorded in the fluorescein channel. **d**, Histograms showing C4F uptake by TmrAB-containing liposomes in the presence of C4F (30 µM) and either Mg-ATP (10 mM, green) or Mg-ADP (10 mM, red), with or without nanobody. **e**, Quantification of peptide transport demonstrates that nanobody binding does not significantly affect TmrAB transport activity. Means ± s.d. are shown (n = 3 technical replicates).

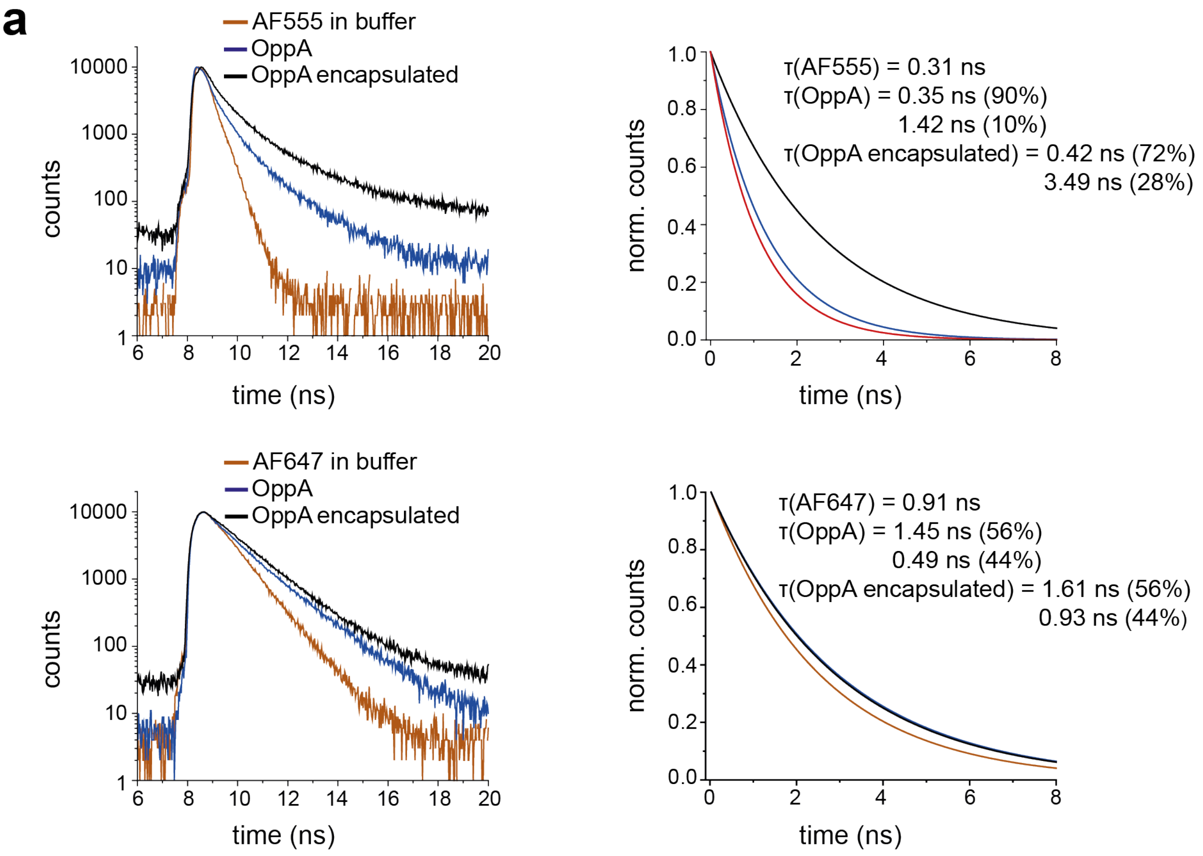

b

Fluorescence anisotropy

	AF555	AF647
free dye	0.11	0.27
OppA	0.17	0.27
OppA encapsulated	0.16	0.27

Supplementary Fig. 8 | Encapsulation does not alter the fluorescence properties of labeled OppA. a, Time-correlated single-photon counting (TCSPC) analysis. Left: TCSPC histogram; Right: normalized fluorescence decay curves for AF555 (top) and AF647 (bottom) under three conditions: free dye in buffer (red), fluorophore-labeled OppA in buffer (OppA$^{AF555/AF647}$, blue), and OppA encapsulated in liposomes (black). Fluorescence lifetimes remained consistent across conditions, indicating that labeling and encapsulation do not affect the dye photophysics. **b**, Steady-state fluorescence anisotropy analyses of AF555 and AF647 measured under the same three conditions as in (a). Anisotropy values remained unchanged, demonstrating that rotational freedom and photophysical behavior of the dyes are preserved upon liposome encapsulation.

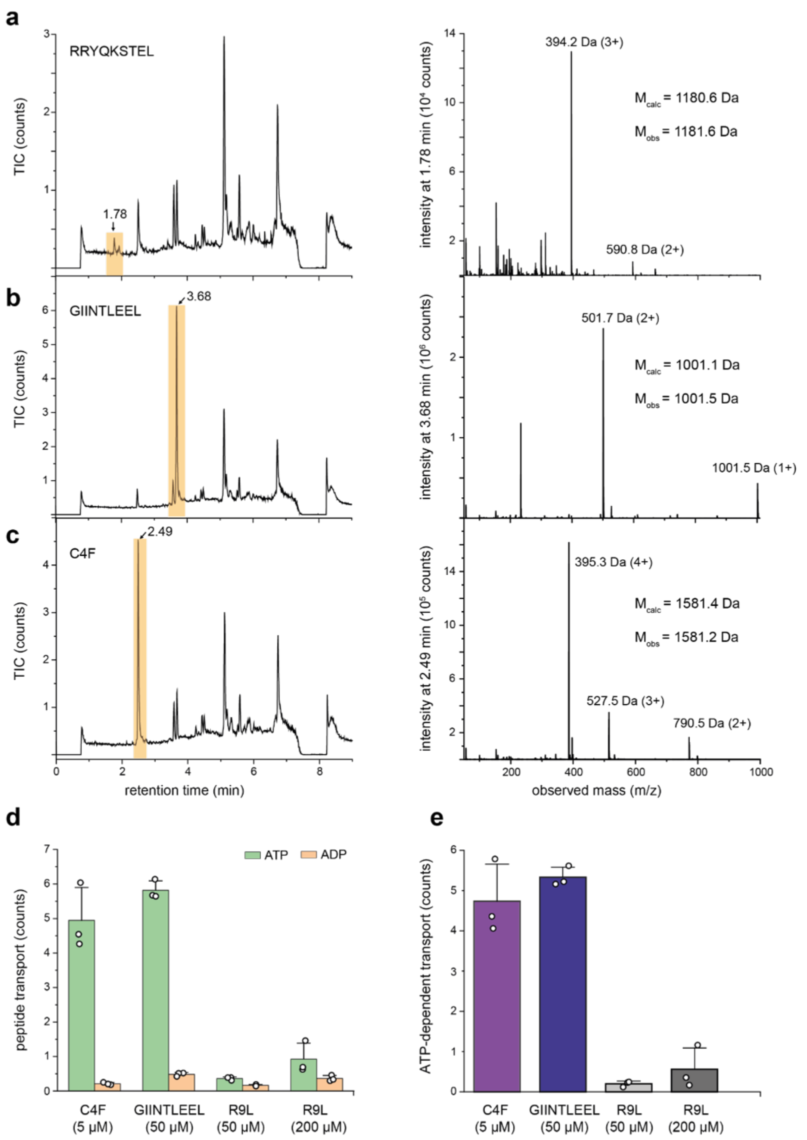

Supplementary Fig. 9 | ATP-dependent transport of unmodified peptides analyzed by LC-MS. Transport was performed with TmrAB^{WT} at 45 °C for 45 min in the presence of Mg-ATP (3 mM) and an ATP regeneration system. **a**, Left: Reverse-phase HPLC (RP-HPLC) profile of the peptide RRYQKSTEL. The total ion current (TIC) at the peptide-specific retention time of 1.78 min was used for quantification (highlighted in yellow orange). Right: Mass spectrum at 1.78 min showing the calculated mass (M_{calc}), doubly charged (2+), and triply charged (3+) species. **b**, Left: RP-HPLC profile of the peptide GIINTLEEL with TIC at 3.68 min (yellow orange) used for quantification. Right: Corresponding mass spectrum at 3.68 min. **c**, Left: RP-HPLC profile of fluorescent peptide RRYC^{Fluorescein}KSTEL (C4F). TIC at 2.49 min (yellow orange) used for quantification. Right: Corresponding mass spectrum at 2.49 min. **d**, All three peptides show significant ATP-dependent transport. **e**, Quantification of ATP-dependent transport by TmrAB after subtraction of background signal form ADP controls. Data are represented as mean± s.d. (*n* = 3 technical replicates).

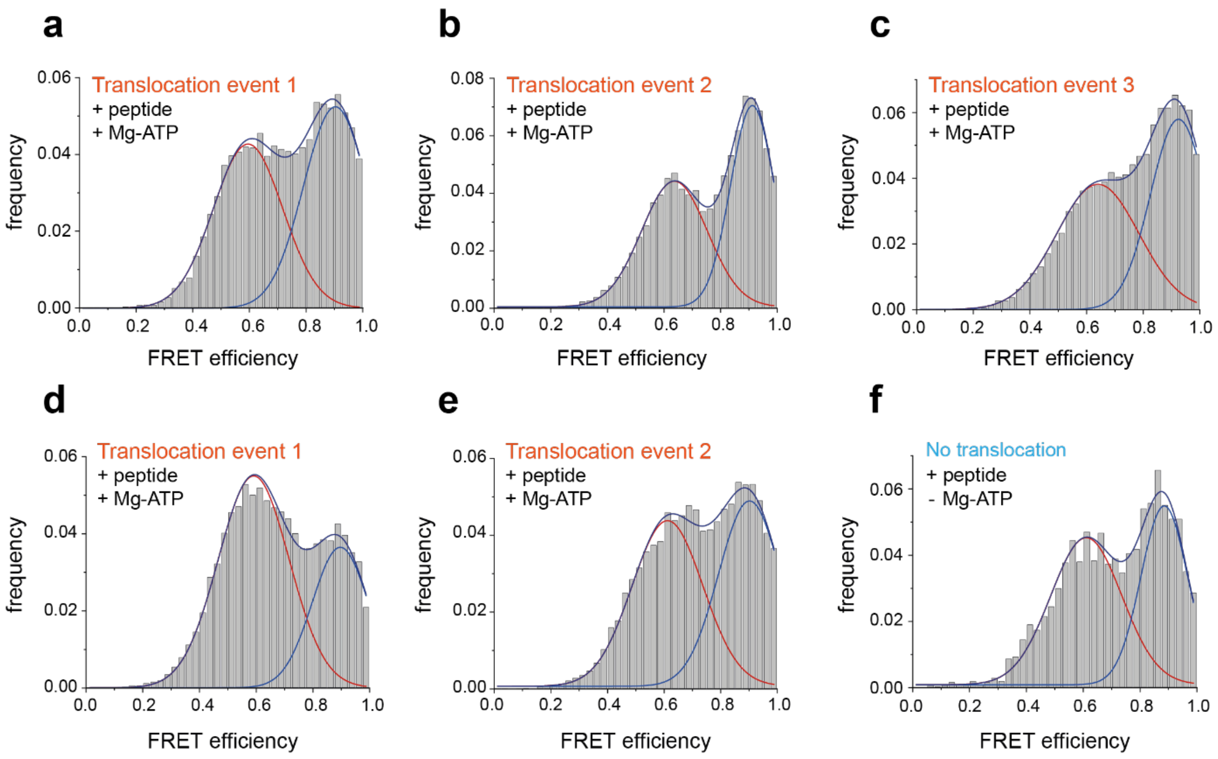

Supplementary Fig. 10 | Single transport events of TmrA^{EQ}B under varying conditions. a, First single-peptide translocation recorded after incubation at 45 °C for 5 min in the presence of 200 µM RRYQKSTEL, 3 mM Mg-ATP, and 10 µM valinomycin (*n* = 148). Liposomes were supplemented with the ionophore in buffer containing potassium ions to dissipate membrane potential. **b**, Second translocation event under identical condition as in (**a**) (*n* = 218). **c**, Third translocation event under identical conditions as in (**b**) (*n* = 180). **d**, First single-peptide translocation at reduced temperature (30 °C) in the presence of 200 µM RRYQKSTEL and 3 mM Mg-ATP, incubated for 5 min (*n* = 460). **e**, Second peptide translocation event under the same conditions as in (**d**) (*n* = 343). **f**, Control experiment performed following the first translocation event. Proteoliposomes were incubated with 200 µM RRYQKSTEL in the absence of Mg-ATP for 5 min at 45 °C. After washing, no second translocation event was observed (*n* = 81 molecules), confirming the ATP dependence of transport.

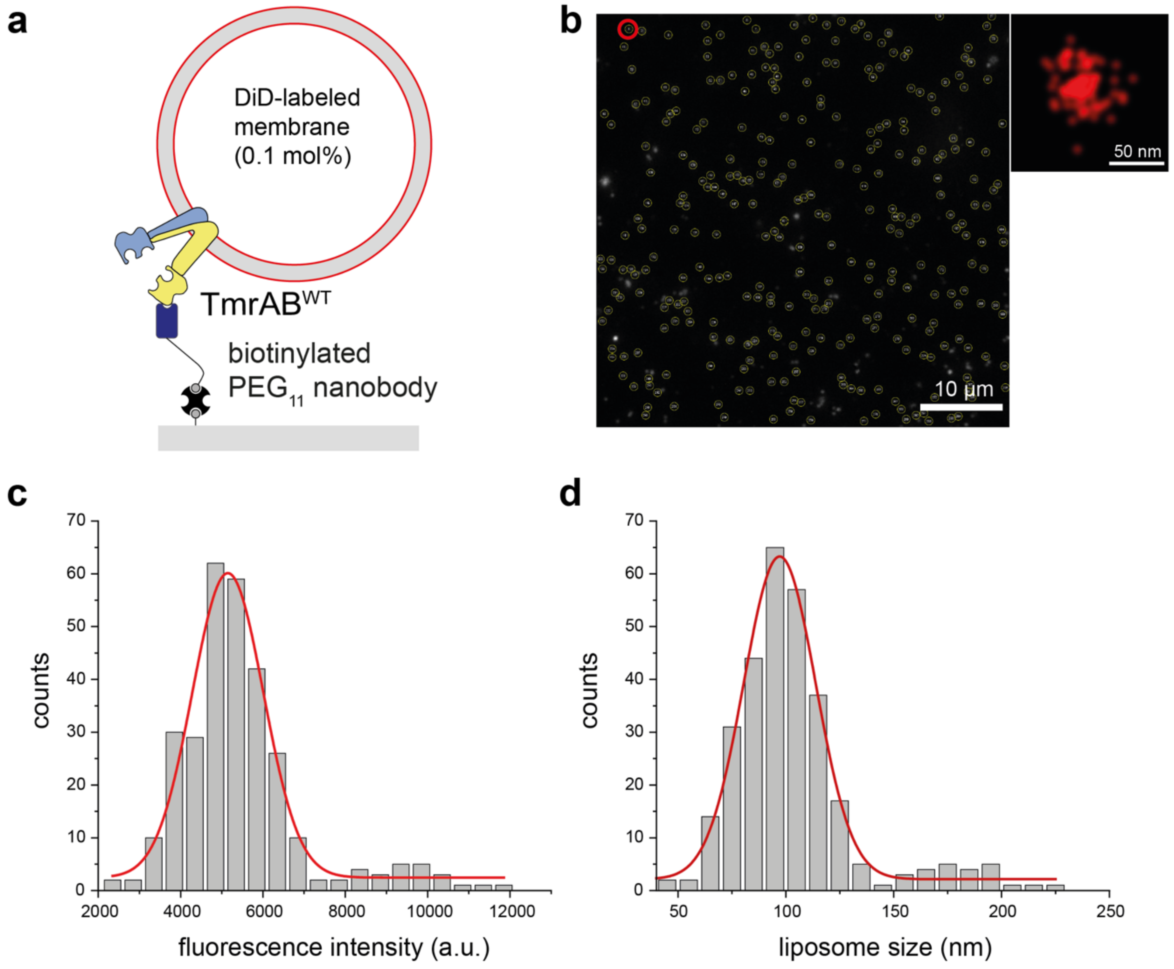

Supplementary Fig. 11 | Homogeneous liposome size distribution. a, Schematic illustration of $TmrAB^{WT}$-containing liposomes immobilized via a biotinylated PEG_{11}-conjugated nanobody. Lipid membranes were stained with the lipophilic carbocyanine dye DiD (0.1 mol%). **b**, Representative *d*STORM field of view showing DiD-stained liposomes. Fluorescence intensities were quantified by integrating the signal within the orange circles (left). A zoom-in of an individual liposome with a diameter of ~100 nm is shown on the right. **c**, Histogram of fluorescence intensities of DiD-stained liposomes, confirming a homogeneous distribution on the microscope surface. **d**, Histogram of liposome diameters measured on the microscope surface, revealing that the predominant population of liposomes has a diameter of a ~100 nm (data as in Fig. 3g).

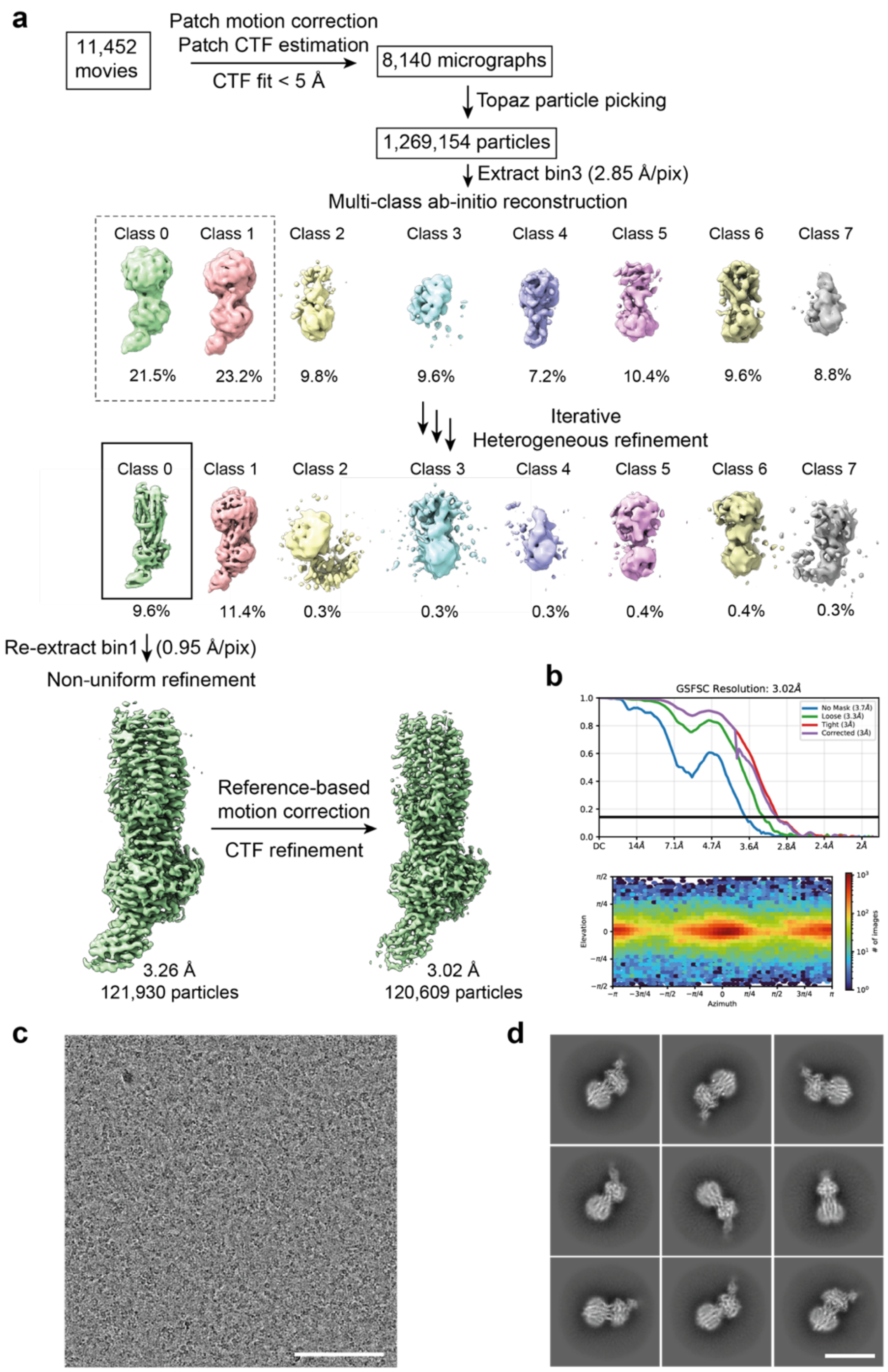

Supplementary Fig. 12 | Cryo-EM analysis of TmrAB^{WT} reconstituted in lipid nanodiscs. a, Cryo-EM data-processing workflow for TmrAB^{WT} in MSP1D1 nanodiscs, resulting in a final refined map at 3.02 Å resolution. **b**, Gold-standard Fourier shell correlation (GSFC) curves for the final refined map (top) and the corresponding particle orientation distribution (bottom). **c**, Representative cryo-EM micrograph of TmrAB^{WT} (scale bar, 100 nm). **d**, Reference-free 2D class averages of TmrAB^{WT} (scale bar, 12 nm).

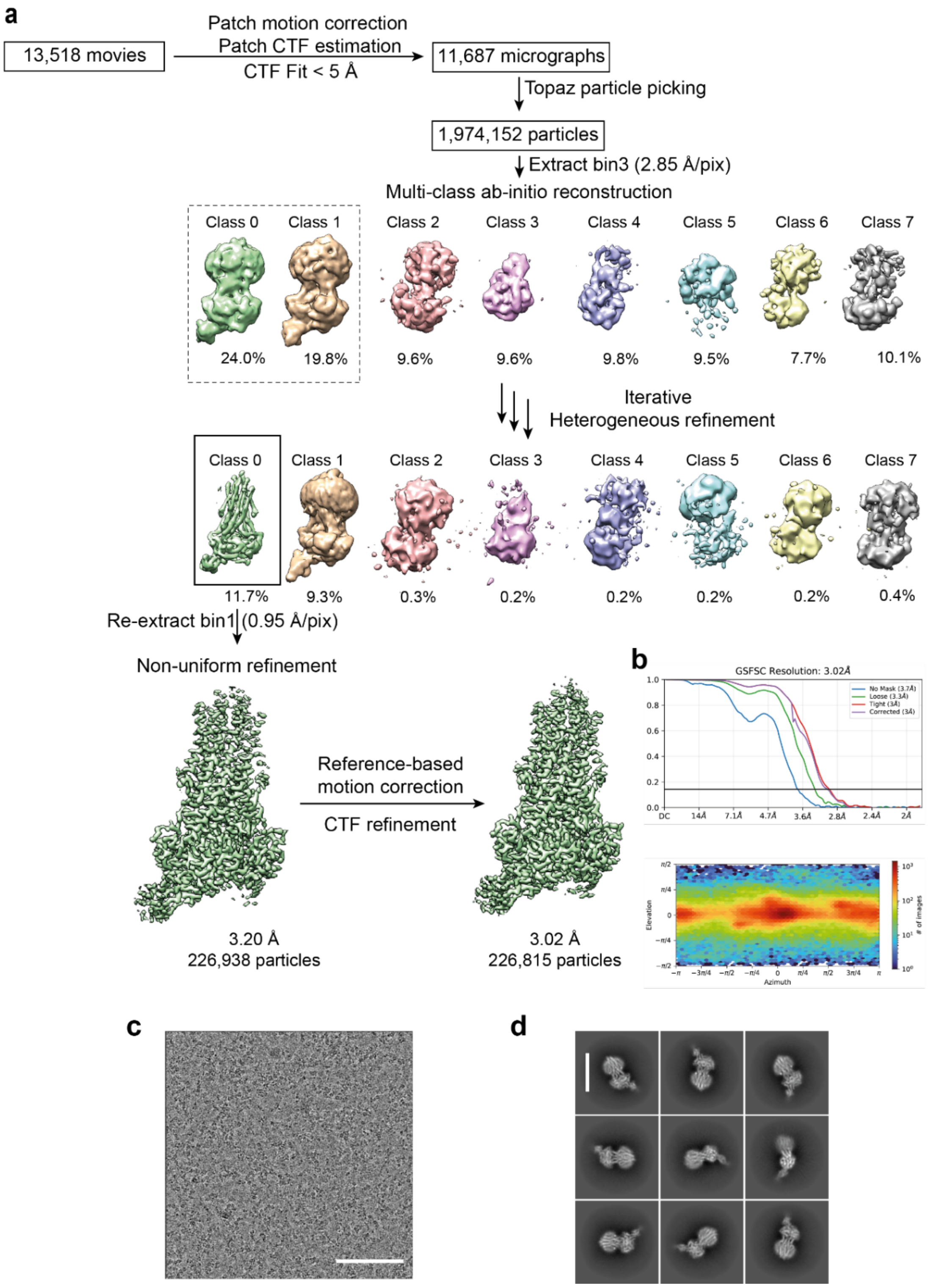

Supplementary Fig. 13 | Cryo-EM analysis of TmrA^{EQ}B reconstituted in lipid nanodiscs. a, Cryo-EM data-processing workflow for TmrA^{EQ}B in MSP1D1 nanodiscs, resulting in a final refined map at 3.02 Å resolution. **b**, Gold-standard Fourier shell correlation (GSFC) curves for the final refined map (top) and the corresponding particle orientation distribution (bottom). **c**, Representative cryo-EM micrograph of TmrA^{EQ}B (scale bar, 100 nm). **d**, Reference-free 2D class averages of TmrA^{EQ}B (scale bar, 12 nm).

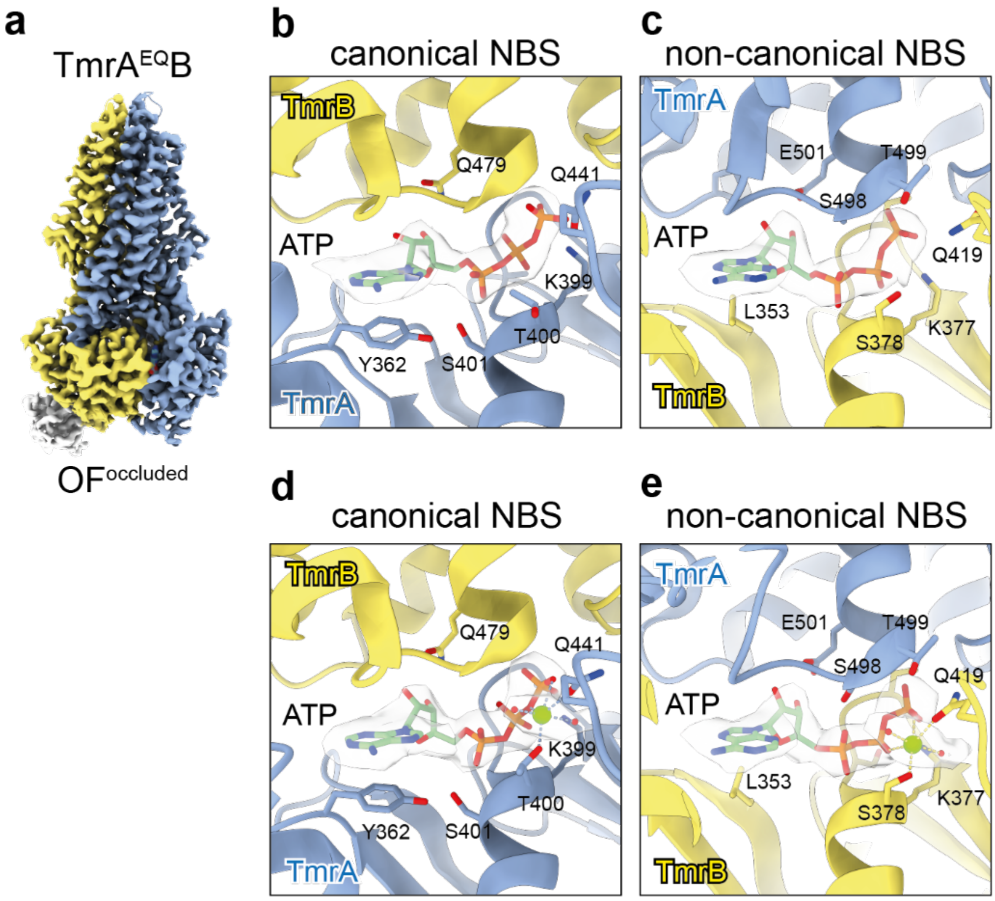

Supplementary Fig. 14 | NBD dimerization and conformational change in TmrA^{EQ}B induced by ATP-EDTA. **a**, Cryo-EM structure of TmrA^{EQ}B in the presence of peptide and ATP-EDTA, revealing a transition to the outward-facing occluded (OF^{occluded}) conformation, resolved at 3.02 Å (EMD-54377, PDB 9RYE). **b**, **c**, Cryo-EM maps confirm ATP binding at both the canonical (**b**) and non-canonical (**c**) nucleotide-binding sites (NBSs), despite the absence of Mg^{2+}. **d**, **e**, Cryo-EM maps of TmrA^{EQ}B bound to Mg-ATP (EMD-4776, PDB 6RAI)[14] at both the canonical (**d**) and non-canonical (**e**) NBSs. Removal of Mg^{2+} (green) has minimal impact on the global structure of the ATP-bound OF^{occluded} state. Subtle local rearrangements are observed in the NBSs, affecting Mg^{2+}-interacting residues (e.g., TmrA^{T400}, TmrB^{Q419}) when Mg^{2+} is absent.

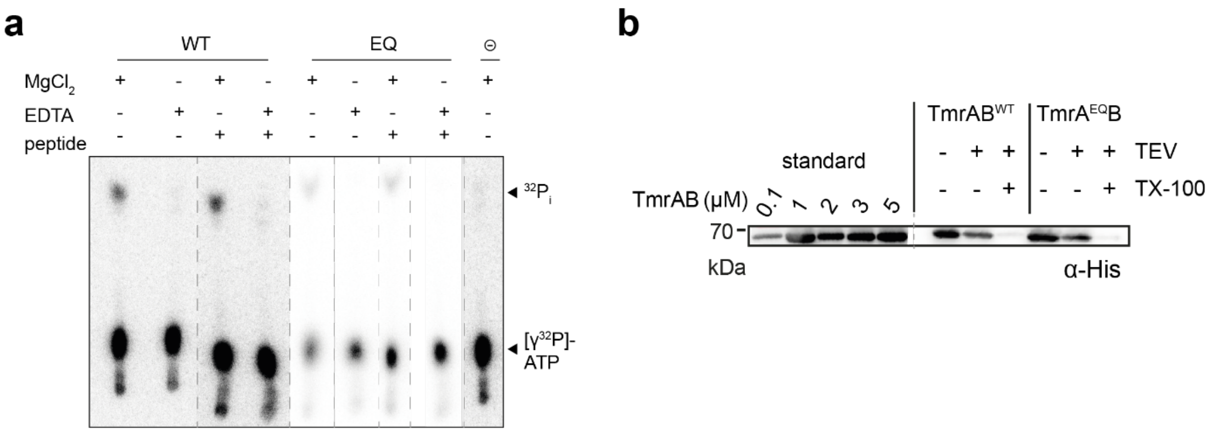

Supplementary Fig. 15 | ATPase activity and membrane orientation of reconstituted TmrAB. **a**, ATPase activity of liposome-reconstituted TmrAB^{WT} and TmrA^{EQ}B incubated with either 5 mM $MgCl_2$ or 10 mM EDTA and 0.3 mM ATP at 45 °C for 15 min, in the presence of 200 µM RRYQKSTEL. [γ^{32}P]-ATP was used to monitor ATP hydrolysis. Representative autoradiograms are shown (corresponding to Fig. 4). The minus icon (–) indicates the autohydrolysis control in the absence of TmrAB. Thin-layer chromatography (TLC) plates were cut along the grey dotted line and merged for clarity. **b**, Membrane orientation of TmrAB determined by a protease protection assay. Cleavage of the C-terminal His-tag at TmrA by Tobacco Etch Virus (TEV) protease was detected using an α-His antibody coupled to horseradish peroxidase (HRP). Uptake-competent orientation was observed in 77% of TmrAB^{WT} and 76% of TmrA^{EQ}B proteoliposomes. Detergent-solubilized TmrAB^{WT} at known concentrations was used to calibrate the anti-His immunoblot and estimate the amount of uptake-competent TmrAB. Membrane was cut as indicated by the grey dashed line. Quantification was performed by band intensity analysis using ImageJ.

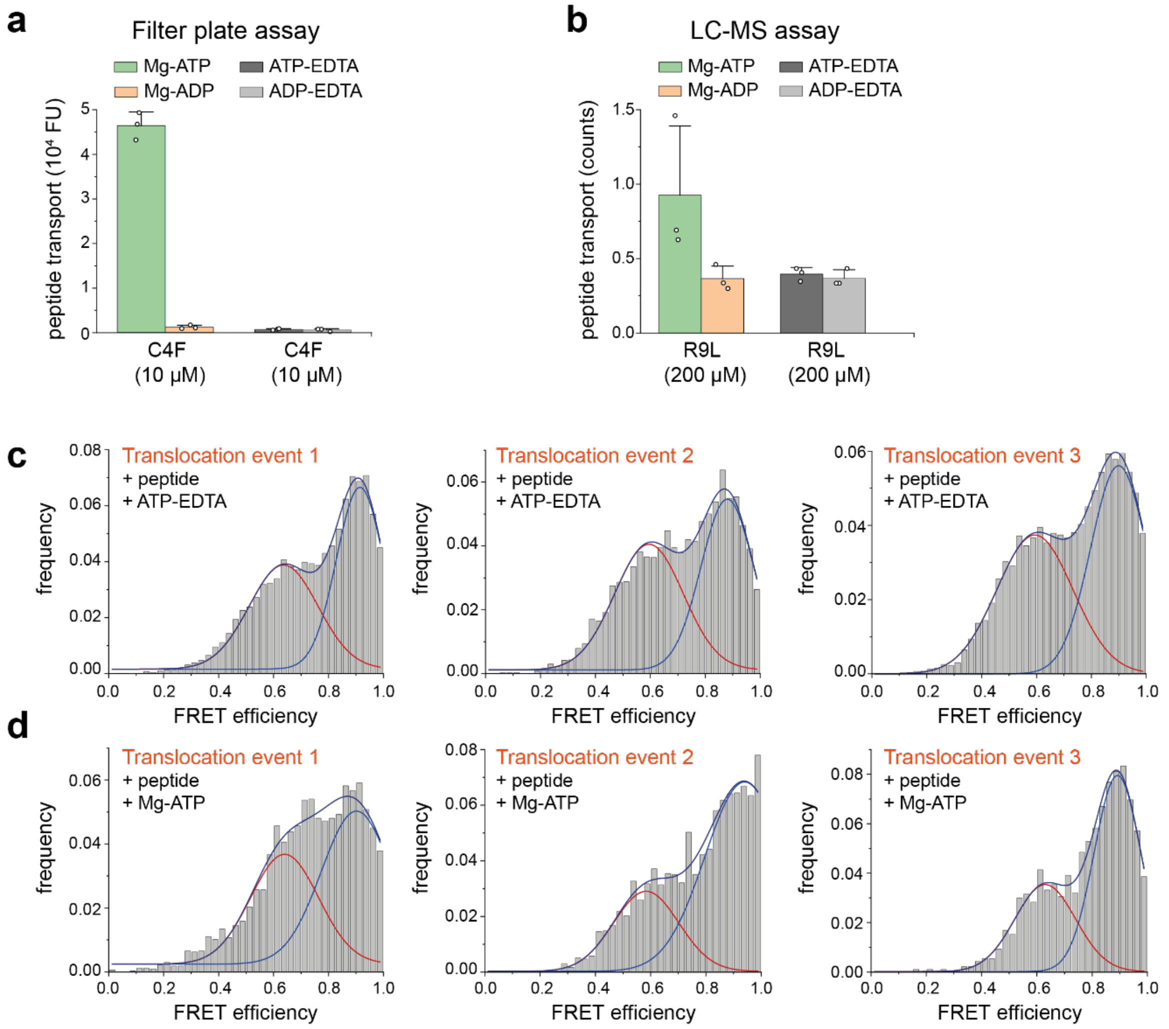

e

	TmrA^EQ^B ATP-EDTA		TmrAB^WT^ ATP-EDTA	
Peptide incubation	Closing rate (s^{-1})	Lumenal peptide concentration (µM)	Closing rate (s^{-1})	Lumenal peptide concentration (µM)
1	2.5 ± 0.7	3 ± 2	2.5 ± 0.2	3 ± 1
2	3.2 ± 0.4	6 ± 2	4.5 ± 1.1	10 ± 4

	TmrA^EQ^B Mg-ATP	
Peptide incubation	Closing rate (s^{-1})	Lumenal peptide concentration (µM)
2	3.7 ± 0.5	7 ± 2
3	4.2 ± 1.0	9 ± 3

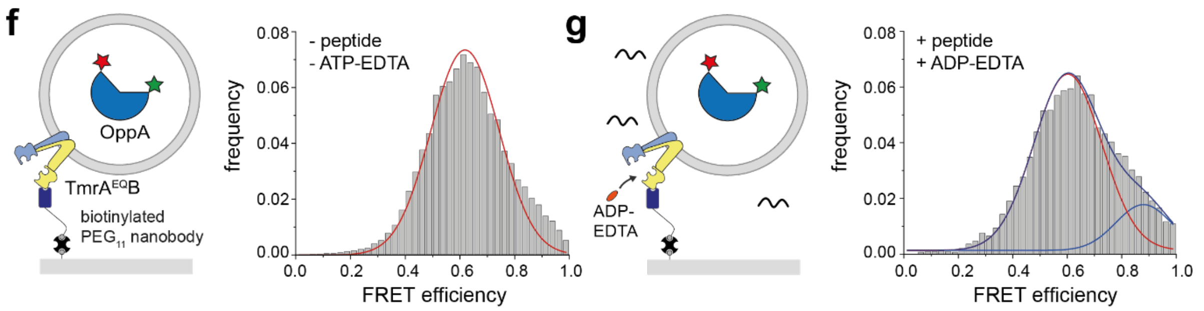

Supplementary Fig. 16 | Consecutive TmrAB transport activity is abolished in the absence of Mg^{2+}. a, Filter plate assay with TmrAB^{WT}-containing proteoliposomes in the presence of C4F (10 µM) and ATP-EDTA (3 mM) shows no transport above ADP-EDTA (3 mM) background, in

contrast to robust transport observed with Mg-ATP (3 mM). **b**, LC-MS-based transport assay with TmrAB^{WT} proteoliposomes confirms only background-level peptide transport under ATP-EDTA or ADP-EDTA conditions (3 mM each) with 200 µM RRYQKSTEL. **c**, Single-molecule translocation assay using TmrAB^{WT} with 200 µM RRYQKSTEL and 3 mM ATP-EDTA at 45 °C for 5 min. Histograms represent translocation event 1 *n* = 158 (left); translocation event 2 *n* = 90 (middle), and translocation event 3 *n* = 246 (right) molecules. The histograms represent three independent experiments. **d**, Positive control: peptide translocation by TmrAB^{WT} under standard conditions (3 mM Mg-ATP and 200 µM RRYQKSTEL, 5 min at 45 °C), with histograms representing translocation event 1 *n* = 54 (left), translocation event 2 *n* = 41 (middle), and translocation event 3 *n* = 27 molecules. **e**, OppA closing rates calculated from dynamic smFRET traces for transport by TmrA^{EQ}B and TmrAB^{WT} in the presence of ATP-EDTA. HMM statistics for TmrA^{EQ}B with ATP-EDTA: translocation event 1 (peptide incubation 1) *n* = 329 (corresponding to data shown in Fig. 5a) and peptide incubation 2 *n* = 323 (data in Fig. 5b). TmrAB^{WT} with ATP-EDTA: translocation event 1 (peptide incubation 1) *n* = 36 and peptide incubation 2 *n* = 116 (data in Supplementary Fig. 16c). For translocation event 1, TmrA^{EQ}B was incubated with ATP-EDTA following peptide incubation with Mg-ATP: peptide incubation 2, *n* = 270 (data in Fig. 5c) or Mg-ATP and peptide incubation after two ATP-EDTA translocations (peptide incubation 3, *n* = 238 (data in Fig. 5d)). **f**, In the absence of peptide and ATP-EDTA, the FRET sensor remains in a low-FRET state, indicating no peptide transport by TmrA^{EQ}B (*E* = 0.6; *n* = 486 molecules). **g**, No peptide transport is observed for TmrA^{EQ}B following a 5 min incubation at 45 °C with 200 µM RRYQKSTEL and 3 mM ADP-EDTA (*n* = 187). Histograms represent three independent experiments.

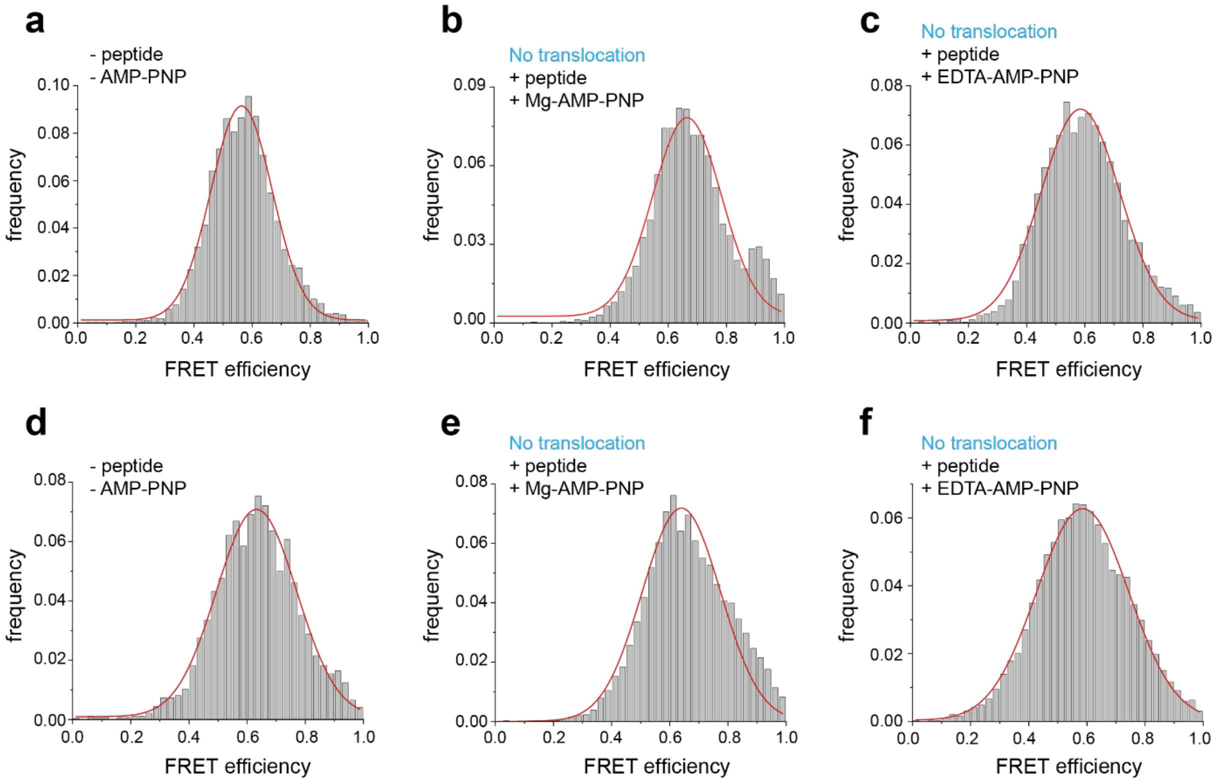

Supplementary Fig. 17 | Influence of the ATP analog AMP-PNP on peptide transport. a, TmrAB^{WT} liposomes in the absence of peptide and AMP-PNP exhibit a low-FRET state of OppA (n = 81). **b**, Peptide translocation assay after incubation at 45 °C for 5 min with 200 µM RRYQKSTEL and 3 mM Mg-AMP-PNP shows no peptide transport via TmrAB^{WT} (n = 96). **c**, Incubation with 200 µM RRYQKSTEL and 3 mM EDTA-AMP-PNP at 45 °C for 5 min also shows no peptide transport by TmrAB^{WT} (n = 116). **d**, Liposomes containing TmrA^{EQ}B recorded in the absence of peptide and AMP-PNP (n = 49). **e**, TmrA^{EQ}B shows no peptide transport after incubation with 200 µM RRYQKSTEL and 3 mM Mg-AMP-PNP for 5 min at 45 °C (n = 149). **f**, Incubation with 200 µM RRYQKSTEL and 3 mM EDTA-AMP-PNP at 45 °C for 5 min does not induce peptide transport by TmrA^{EQ}B (n = 181).

Supplementary Table 1. Cryo-EM data collection, refinement and validation statistics.

	TmrAB^{WT}<br>EMD-54378	TmrA^{EQ}B<br>EMD-54377
**Data collection and processing**		
Magnification	150,000x	150,000x
Voltage (kV)	200	200
Electron exposure (e–/Å^2)	28.3	28.3
Defocus range (μm)	-1.2 to -1.8	-1.2 to -1.8
Pixel size (Å)	0.95	0.95
Symmetry imposed	C1	C1
Initial micrographs (no.)	11,452	13,712
Final micrographs (no.)	8,140	11,687
Initial particle images (no.)	1,269,154	1,974,152
Final particle images (no.)	120,609	226,815
Map resolution (Å)	3.02	3.02
FSC threshold	0.143	0.143
Map sharpening *B* factor (Å^2)	-115.1	-147.3

	TmrAB^{WT}<br>EMD-54378<br>PDB 9RYF	TmrA^{EQ}B<br>EMD-54377<br>PDB 9RYE
**Refinement**		
Model composition		
Non-hydrogen atoms	10,168	10,168
Protein residues	1,279	1,279
ATP/ADP	2	2
B factors (Å^2)		
Protein	70.65	96.30
Ligand	53.23	78.33
R.m.s. deviations		
Bond lengths (Å)	0.004	0.004
Bond angles (°)	0.586	0.611
**Validation**		
MolProbity score	1.68	1.63
Clashscore	7.68	6.41
Poor rotamers (%)	0.00	0.00
Ramachandran plot		
Favored (%)	96.23	95.99
Allowed (%)	3.77	4.01
Disallowed (%)	0.00	0.00
